# Transcriptomic analyses of murine ventricular cardiomyocytes

**DOI:** 10.1038/sdata.2018.170

**Published:** 2018-08-21

**Authors:** Morgan Chevalier, Sarah H. Vermij, Kurt Wyler, Ludovic Gillet, Irene Keller, Hugues Abriel

**Affiliations:** 1 Ion Channels and Channelopathies Laboratory, Institute for Biochemistry and Molecular Medicine, University of Bern, 3012 Bern, Switzerland; 2 Interfaculty Bioinformatics Unit and Swiss Institute of Bioinformatics, University of Bern, 3012 Bern, Switzerland; 3 Pain Center, Department of Anesthesiology, University Hospital Center (CHUV) and Faculty of Biology and Medicine (FBM), University of Lausanne, 1011 Lausanne, Switzerland; 4 Department of Fundamental Neurosciences, Faculty of Biology and Medicine (FBM), University of Lausanne, 1005 Lausanne, Switzerland; 5 Department for BioMedical Research and Swiss Institute of Bioinformatics, University of Bern, 3007 Bern, Switzerland

**Keywords:** Gene expression, Mechanisms of disease

## Abstract

Mice are used universally as model organisms for studying heart physiology, and a plethora of genetically modified mouse models exist to study cardiac disease. Transcriptomic data for whole-heart tissue are available, but not yet for isolated ventricular cardiomyocytes. Our lab therefore collected comprehensive RNA-seq data from wildtype murine ventricular cardiomyocytes as well as from knockout models of the ion channel regulators CASK, dystrophin, and SAP97. We also elucidate ion channel expression from wild-type cells to help forward the debate about which ion channels are expressed in cardiomyocytes. Researchers studying the heart, and especially cardiac arrhythmias, may benefit from these cardiomyocyte-specific transcriptomic data to assess expression of genes of interest.

## Background & Summary

**Figure 1:**
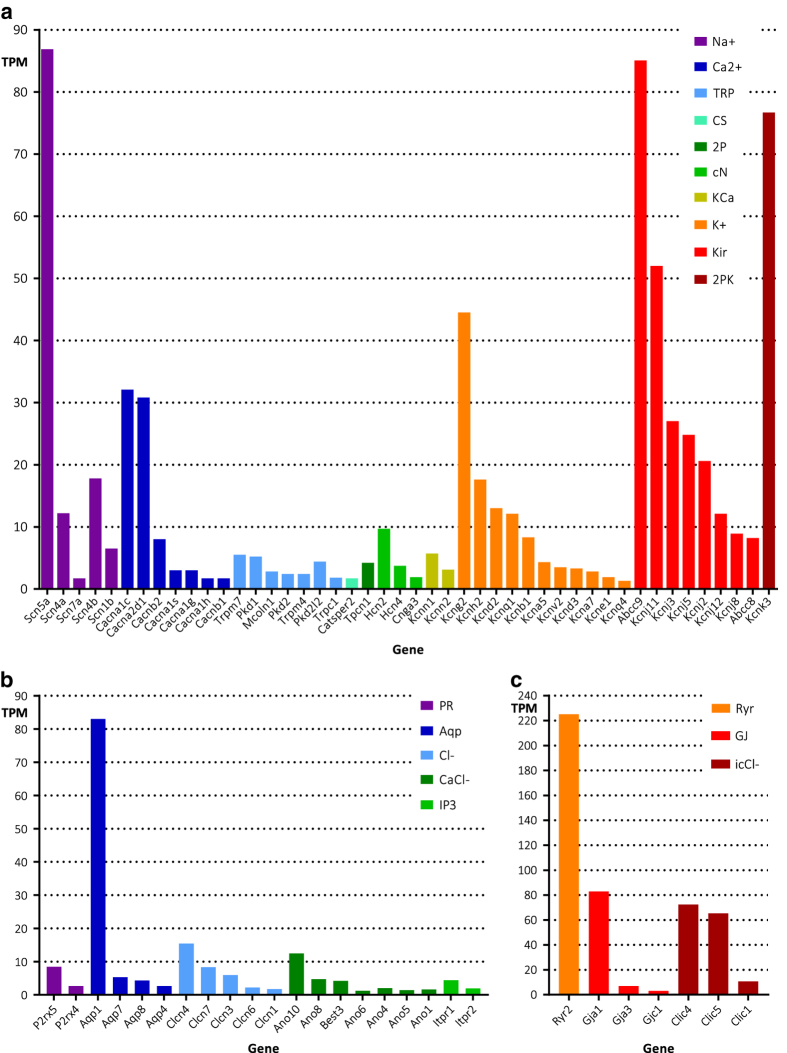
Gene expression of ion channels in murine ventricular cardiomyocytes. (**a**) Expression levels of voltage-gated ion channel genes: voltage-gated sodium channels (Na+; purple), voltage-gated calcium channels (Ca2+; blue), transient receptor potential cation channels (TRP; light blue), CS, CatSper channels (aqua), two-pore channels (2P; green), cyclic-nucleotide-regulated channels (cN; light green), calcium-activated potassium channels (KCa; ochre), voltage-gated potassium channels (K+; orange), inwardly rectifying potassium channels (Kir; red) and two-pore potassium channels (2PK; burgundy). (**b**) Expression levels of the ligand-gated purinergic receptor gene (PR; purple) and of ion channel genes from the “other” category: aquaporins (Aqp; blue), voltage-sensitive chloride channels (Cl-; light blue), calcium-activated chloride channels (CaCl-; green) and inositol triphosphate receptors (IP3; light green). (**c**) Expression levels of more ion channel genes from the “other” category: ryanodine receptors (Ryr; orange), gap junction proteins (GJ; red) and chloride intracellular channels (icCl-; burgundy). All expression levels are average TPM values of WT samples (*n*=5). Shown are genes with more than 75 reads per gene (normalized for gene length, prior to conversion to TPM) from Tables 1, Table 2 and Table 3 (available online only).

In this study, we present next-generation RNA sequencing (RNA-seq) data of murine ventricular cardiomyocytes (CMC). To date, only whole-heart RNA-seq data have been published^1–3^, in which a variety of cell types, such as fibroblasts, endothelial cells, and atrial and ventricular cardiomyocytes, are pooled. We endeavoured to provide RNA-seq data of isolated CMCs for several reasons. Firstly, since the pump function of the heart relies on proper CMC function, CMCs are the most thoroughly studied cardiac cell type. Researchers studying CMCs may benefit from CMC-specific RNA-seq data from which expression of genes of interest can be extracted. Secondly, because of the crucial role of ion channels in cardiac electrical excitability and arrhythmogenesis, researchers that study cardiac arrhythmias have debated the question of which ion channels are expressed in CMCs. However, existing ion channel expression data are low-throughput, often contradictory^4–6^, fragmented^7^, or expression is assessed in the whole heart. The present work reveals the expression of the more than 350 ion channel family members, including pore-forming and auxiliary subunits, in CMCs (see Fig. 1 and Tables 1, Tables 2 and Tables 3 (available online only)). We therefore believe that these data will be valuable for ion channel researchers attempting to resolve the ongoing debate.

We have also included cardiac-specific knockout models of the ion channel regulators dystrophin, synapse-associated protein-97 (SAP97), and calmodulin-activated serine kinase (CASK). They interact with ion channels and modify their cell biological properties, such as membrane localization^3,8–11^. Notably, CASK provides a direct link between ion channel function and gene expression. It regulates transcription factors (TFs) in the nucleus, such as Tbr-1, and induces transcription of T-element-containing genes^12^. CASK also regulates TFs of the basic helix-loop-helix family, which bind E-box elements in promoter regions, by modulating the inhibitor of the DNA-binding-1 TF^13^. Additionally, CASK and SAP97 directly interact with each other^11^. For these reasons, we include CASK, SAP97, and dystrophin knockout mice to investigate whether these three proteins have a similar effect on gene expression, which may suggest their involvement in similar pathways. However, research beyond the scope of this paper would be needed to determine whether CASK-dependent TF regulation caused the differential expression that we observed.

To date, mutations in approximately 27 ion channel genes have been associated with cardiac arrhythmias, such as congenital short- and long-QT syndrome (SQTS and LQTS), Brugada syndrome (BrS), and conduction disorders (see http://omim.org)^14–16^. Notably, our ion channel expression data, as presented in Fig. 1 and Tables 1, Table 2 and Table 3 (available online only), reveal that several arrhythmia-associated ion channel genes are not or are scarcely expressed in murine ventricular CMCs (including Kcne2, Kcne3, Scn2b, and Scn3b). Although murine and human ion channel expression may differ, we are presently unaware of any available transcriptome of human CMCs^17,18^. We are also unable to either exclude or assess the effect of enzymatic isolation on the transcriptome. Finally, other cardiac cell types such as (myo)fibroblasts may express these ion channels and therefore may be important for arrhythmogenesis. Indeed, many ion channel genes that are not expressed in cardiomyocytes have been reported in murine whole-heart tissue^2^. These include Scn1a, Scn3b, 10 voltage-gated Ca^2+^ channels, 10 K_v_ channels, and four two-pore K^+^ channels. Conversely, all ion channel genes expressed in CMCs are also reported in whole-heart expression data.

**Figure 2:**
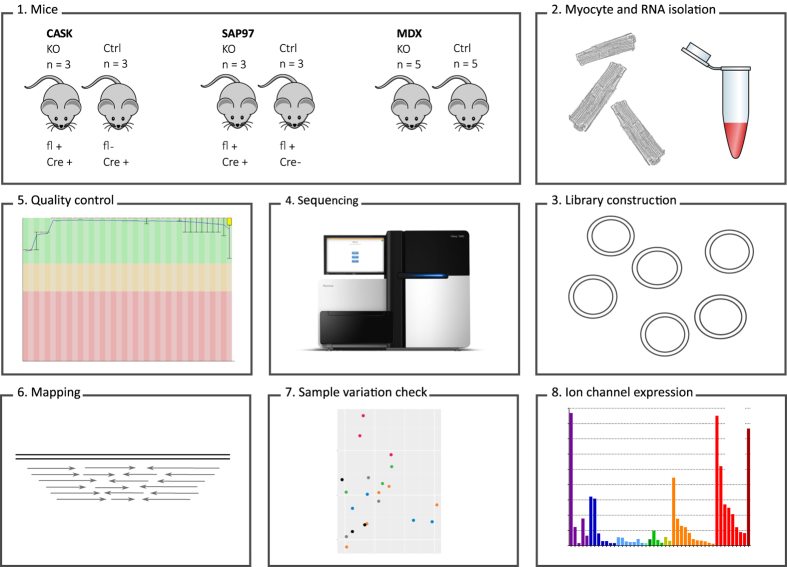
Experimental design and workflow. (1) 22 mice with six different genetic backgrounds (CASK KO and control, SAP97 KO and control, and MDX and control) were used. fl+, first exon of gene is floxed; Cre+, Cre recombinase is expressed. (2) Cardiomyocytes were isolated on a Langendorff system and RNA was isolated with a FFPE Clear RNAready kit. (3) Libraries were constructed with 1 μg RNA per sample using a TrueSeq Stranded Total RNA protocol and (4) sequenced on an Illumina HiSeq3000 machine. (5) Quality of the reads was assessed with FastQC, and (6) reads were mapped to the *Mus musculus* reference genome (GRCm38.83) with Tophat. (7) To assess sample variation within each group, we performed principle component analyses (PCA) (see Fig. 3). (8) Lastly, ion channel expression was determined.

In sum, this study presents RNA-seq data from wildtype murine ventricular CMCs, as well as from SAP97, CASK, and dystrophin knockouts and controls (see Fig. 2 for a schematic overview of study design). We performed differential gene expression analysis to compare the knockouts to their controls, and we extracted wildtype ion channel gene expression data (Tables 1, Table 2 and Table 3 (available online only), Fig. 1). We believe that these data will be valuable for researchers studying cardiomyocytes and ion channels to assess expression of genes of interest.

## Methods

### Mouse models

All animal experiments conformed to the *Guide to the Care and Use of Laboratory Animals* (US National Institutes of Health, publication No. 85-23, revised 1996); have been approved by the Cantonal Veterinary Administration, Bern, Switzerland; and have complied with the Swiss Federal Animal Protection Law. Mice were kept on a 12-hour light/dark cycle. Lights were on from 6:30 AM to 6:30 PM. To avoid the influence of circadian rhythm, mice were sacrificed between 10:00 AM and 1:00 PM. Mice were all male and were between the ages of 8 and 15 weeks.

#### MHC-Cre

The cardiac-specific murine alpha-myosin heavy chain (μMHC) promoter drives the expression of Cre recombinase, which, in turn, can recombine LoxP sequences. The μMHC-Cre strain was generated as previously described^19^ and acquired from the Jackson Laboratory (stock #011038).

#### CASK and SAP97 knockout mice

CASK KO and SAP97/Dlg1 KO mice were generated as previously described^9,20^. Both the CASK and SAP97 mouse lines were on mixed backgrounds. The appropriate control mice were selected in accordance with the publications that characterized both mouse lines^9,20^. CASK control mice express Cre while the first CASK exon is not floxed. SAP97 control mice are Cre-negative and the first SAP97 gene was floxed.

#### Dystrophin knockout (MDX-5CV) mice

The MDX-5CV strain demonstrates total deletion of the dystrophin protein. It was created as previously described^21^, and acquired from the Jackson laboratory (stock #002379). MDX mice were on pure Bl6/Ros backgrounds. Control mice were on pure Bl6/J background, except for MDX_Ct5 and MDX_5, which were Bl6/Ros mice backcrossed three times on Bl6/J.

### Cardiomyocyte isolation

Mice (*n*=3–5 per genotype, male, age 10–15 weeks) were heparinized (intraperitoneal injection of 100 μL heparin (5000 U/mL; Biochrom AG)) and killed by cervical dislocation. Hearts were excised, and the aortas were cannulated in ice-cold phosphate-buffered saline (PBS). Subsequently, hearts were perfused on a Langendorff system in a retrograde manner at 37 °C with 5 mL perfusion buffer (1.5 mL/min; in mM: 135 NaCl, 4 KCl, 1.2 NaH_2_PO_4_, 1.2 MgCl_2_, 10 HEPES, 11 glucose), followed by the application of type II collagenase (Worthington CLS2; 25 mL of 1 mg/mL in perfusion buffer with 50 μM CaCl_2_). Left and right ventricles were triturated in PBS to dissociate individual ventricular cardiomyocytes and then filtered through a 100 μm filter.

### RNA extraction and sequencing

RNA-seq was performed by the Next Generation Sequencing Platform at the University of Bern. Total RNA was isolated from freshly dissociated cardiomyocytes with an FFPE Clear RNAready kit (AmpTec, Germany), which included a DNase treatment step. RNA quality was assessed with Qubit and Bioanalyzer, and RNA quantity was checked with Qubit.

To allow sequencing of long non-coding RNA (lncRNA), libraries were constructed with 1 μg RNA using the TruSeq Stranded Total RNA kit after Ribo-Zero Gold (Illumina) treatment for rRNA depletion. Library molecules with inserts <300 base pairs (bp) were removed. Paired-end libraries (2x150 bp) were sequenced on an Illumina HiSeq3000 machine.

### RNA-seq data analysis

Between 17.5 and 56.4 million read pairs were obtained per sample and the quality of the reads was assessed using FastQC v.0.11.2 (http://www.bioinformatics.babraham.ac.uk/projects/fastqc/). Ribosomal RNA (rRNA) was removed by mapping the reads with Bowtie2 v.2.2.1 (ref. 22) to a collection of rRNA sequences (NR_003279.1, NR_003278.3 and NR_003280.2) downloaded from NCBI (www.ncbi.nlm.nih.gov). No quality trimming was required.The remaining reads were mapped to the *Mus musculus* reference genome (GRCm38.83) with Tophat v.2.0.13 (ref. 23). We used htseq-count v.0.6.1 (ref. 24) to count the number of reads overlapping with each gene, as specified in the Ensembl annotation (GRCm38.83). Detailed information about the genes including the Entrez Gene ID, the MGI symbol and the description of the gene was obtained using the Bioconductor package BioMart v.2.26.1 (ref. 25).

Raw reads were corrected for gene length and TPM (transcripts per million) values were calculated to compare the expression levels among samples. Gene lengths for the latter step were retrieved from the Ensembl annotation (GRCm38.83) as the total sum of all exons.

Principal component analysis (PCA) plots were done in DESeq2 v.1.10.1 (ref. 26) (https://bioconductor.org/packages/release/bioc/html/DESeq2.html) using the 500 genes with the most variable expression across samples. A regularized log transformation was applied to the counts before performing the PCA.

### Statistics

To assess differential gene expression between genotypes, a Wald test was performed with the Bioconductor package DESeq2 v.1.10.1 (ref. 26). We considered *p* values of up to 0.01, accounting for a Benjamini-Hochberg false discovery rate adjustment, to indicate significant difference. Statistical tools used included DESeq2, R-3.2.5 (https://cran.r-project.org), and Biomart_2.26.1 (www.biomart.org).

## Data Records

The data were submitted to NCBI Gene Expression Omnibus (GEO) (Data Citation 1). This GEO project contains raw data and TPM values from all samples, and differential gene expression analysis between knockout and control samples.

## Technical Validation

### RNA metrics

RNA-seq yielded 1.0 billion read pairs in total, with an average of 44.5 million read pairs per sample (standard deviation 8.4 million). The number of read pairs (in millions) was 306 for CASK KO and Ctrl, 268 for SAP97 KO and Ctrl, and 404 for MDX and Ctrl (see Table 4 for an overview of RNA-seq metrics, including mapping rates). One sample (MDX_1) yielded few reads and was therefore excluded from further analyses. The proportion of reads mapping to annotated exons ranged from 65 to 77%. Mapping, no-feature (2–13%), and ambiguous (11–23%) read pairs together accounted for 89–97% of the total number of RNA reads (Table 4). Read pairs covered 49,671 genes of the *Mus musculus* reference genome (GRCm83.38).

### Quality assessment

**Figure 3:**
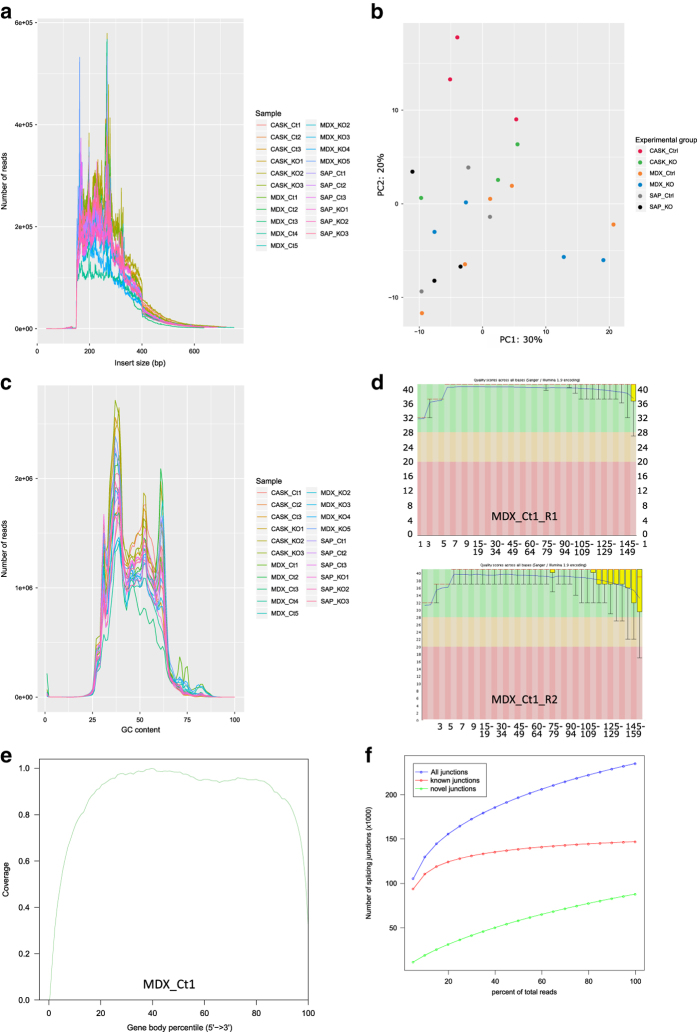
Quality control. (**a**) Histogram of inferred insert size for each sample, which represents distance between the two reads of one RNA fragment. (**b**) Principle component analyses (PCA) plots were performed to assess variability of samples within and between groups. Plot of the first two axes from a PCA based on the 500 genes with the most variable expression across all samples except MDX_1. CASK control (red, *n*=3) and KO (green, *n*=3); MDX control (orange, *n*=5) and KO (blue, *n*=4); SAP97 control (grey, *n*=3) and KO (black, *n*=3). (**c**) Distribution of GC content of the reads for each sample. (**d**) Base quality (Phred scores) along the length of the reads in each FastQC file of MDX_Ct1 as representative sample. The box plots are drawn as follows: red line, median; yellow box, range between upper and lower quartiles; whiskers, range between 10 and 90% quantiles. The blue line shows the mean quality. Y-axis represents quality scores across all bases. X-axis represents position in read (bp). (**e**) Gene body coverage. Distribution of reads along the length of the genes (5’-end on the left, 3’-end on the right). Shown image of sample MDX_Ct1 is representative for all samples. (**f**) Saturation report, depicting the number of splice junctions detected using different subsets of the data from 5 to 100% of all reads. Red, known junction based on the provided genome annotation; green, novel junctions; blue, all junctions. The red line reaches a plateau where adding more data does not increase the number of detected junctions, indicating that the sequencing depth suffices for performing alternative splicing analysis.

The quality of all samples was assessed with FastQC. Except for MDX_1, all samples were of high quality. Where applicable, a representative example (MDX_Ct1) is shown. Firstly, the insert size histogram (Fig. 3a) shows that the inferred insert size of each sample exceeded 150 base pairs, demonstating that the sequencing was not contaminated by adapter sequences. Secondly, the GC content plot (Fig. 3c) ideally shows a roughly normal distribution centred around the average GC content of the genome, which varies between species. The peaks observed in Fig. 3c are likely caused by sequences that are detected at high copy numbers, and should not pose problems for downstream analyses. Furthermore, Phred scores (Fig. 3d) are well within the green area of the graph indicating good base quality along the length of reads. As well, the gene coverage graph (Fig. 3e) of sample MDX_Ct1 shows that reads are distributed evenly along the length of the gene body. Because the gene coverage for all other samples is highly comparable to that of MDX_Ct1, only one example is shown. Lastly, the saturation report (Fig. 3f) represents the number of splice junctions detected using different subsets of the data from 5 to 100% of all reads. At sequencing depths sufficient to perform alternative splicing analysis, at least the red line, representing known junctions, should reach a plateau where adding more data does not much increase the number of detected junctions. Only MDX_1 does not reach this plateau.

### Gene expression variation of biological replicates

We performed Principle Component Analyses (PCA) to assess whether samples from the same experimental group have similar gene expression profiles (Fig. 3b). Of note, samples within each sample group still show considerable variation. The mixed genetic background of most sample groups may explain this variation; only the MDX control mice are on a pure Bl6/J background. The variation seen in MDX control mice is likely due to a batch effect, as two rounds of samples were sequenced. However, considering that PCA plots are based on the 500 genes with the highest variability in one sample, our genes of interest, including all ion channel genes, show similar expression levels throughout all samples.

### Ion channel expression

Based on the list of ion channel genes from HUGO Gene Nomenclature Committee (https://www.genenames.org/cgi-bin/genefamilies/set/177), we distilled ion channel expression from WT mice expressed as TPM (Tables 1, Table 2 and Table 3 (available online only), Fig. 1).

## Additional information

**How to cite this article**: Chevalier, M. *et al*, Transcriptomic analyses of murine ventricular cardiomyocytes. *Sci. Data* 5:180170 doi: 10.1038/sdata.2018.170 (2018).

**Publisher’s note**: Springer Nature remains neutral with regard to jurisdictional claims in published maps and institutional affiliations.

## Supplementary Material



## Figures and Tables

**Table 1:** Expression of voltage-gated ion channels

**Gene**	**Protein**	**TPM**
**Sodium channels, voltage-gated**		
Scn5a	Nav1.5	86.900
Scn4a	Nav1.4	12.163
Scn7a	Nav2.1	1.650
Scn10a	Nav1.8	0.180
Scn3a	Nav1.3	0.123
Scn2a	Nav1.2	0.025
Scn11a	Nav1.9	0.009
Scn8a	Nav1.6	0.004
Scn1a	Nav1.1	0.003
Scn9a	Nav1.7	0.000
Scn4b	β4 subunit	17.765
Scn1b	β1 subunit	6.524
Scn3b	β3 subunit	0.074
Scn2b	β2 subunit	0.041
**Calcium channels voltage-gated**		
Cacna1c	Cav1.2	32.050
Cacna1s	Cav1.1	3.045
Cacna1g	Cav3.1	3.006
Cacna1h	Cav3.2	1.738
Cacna1a	Cav2.1	0.360
Cacna1d	Cav1.3	0.225
Cacna1b	Cav2.2	0.028
Cacna1i	Cav3.3	0.008
Cacna1e	Cav2.3	0.003
Cacna1f	Cav1.4	0.000
Cacna2d1	α2 and δ1 subunit	30.799
Cacna2d3	α2 and δ3 subunit	0.048
Cacna2d4	α2 and δ4 subunit	0.000
Cacnb2	β2 subunit	8.023
Cacnb1	β1 subunit	1.714
Cacnb3	β3 subunit	0.398
Cacnb4	β4 subunit	0.108
Cacng6	γ6 subunit	1.513
Cacna2d2	γ1 subunit	0.455
Cacng7	γ7 subunit	0.261
Cacng1	γ7 subunit	0.024
Cacng4	γ4 subunit	0.010
Cacng5	γ5 subunit	0.008
Cacng2	γ2 subunit	0.002
Cacng3	γ3 subunit	0.000
Cacng8	γ8 subunit	0.000
**Transient receptor potential cation channels**		
Trpm7	TRPM7	5.530
Pkd1	TRPP1	5.248
Pkd2l2	TRPP5	4.380
Mcoln1	TRPML1	2.836
Trpm4	TRPM4	2.431
Pkd2	TRPP2	2.424
Trpc1	TRPC1	1.836
Trpc3	TRPC3	0.875
Trpv2	TRPV2	0.391
Trpv4	TRPV4	0.366
Trpm3	TRPM3	0.287
Trpc6	TRPC6	0.051
Trpm6	TRPM6	0.037
Trpc4	TRPC4	0.033
Trpv1	TRPV1	0.026
Trpa1	TRPA1	0.022
Mcoln3	TRPML3	0.018
Trpv3	TRPV3	0.016
Mcoln2	TRPML2	0.011
Trpm2	TRPM2	0.011
Trpm5	TRPM5	0.005
Trpv5	TRPV5	0.005
Trpv6	TRPV6	0.005
Trpc7	TRPC7	0.002
Pkd2l1	TRPP3	0.000
Trpc2	TRPC2	0.000
Trpc5	TRPC5	0.000
Trpm1	TRPM1	0.000
Trpm8	TRPM8	0.000
**CatSper channels**		
Catsper2	CATSPER2	1.744
Catsper3	CATSPER3, CACRC	0.127
Catsper4	CATSPER4	0.060
Catsperd	δ subunit	0.058
Catsperg1	γ1 subunit	0.031
Catsperb	β subunit	0.003
Catsper1	CATSPER1	0.000
Catsperg2	γ2 subunit	0.000
**Two-pore channels**		
Tpcn1	TPCN1	4.183
Tpcn2	TPCN2	0.446
**Cyclic nucleotide-regulated channels**		
Hcn2	HCN2	9.736
Hcn4	HCN4	3.661
Cnga3	CNGA3	1.880
Cngb3	CNGB3	1.051
Hcn1	HCN1	0.229
Cngb1	CNGB1	0.067
Hcn3	HCN3	0.056
Cnga1	CNGA1	0.000
Cnga2	CNGA2	0.000
Cnga4	CNGA4	0.000
**Potassium channels, calcium-activated**		
Kcnn1	KCa2.1	5.677
Kcnn2	KCa2.2	3.107
Kcnt2	Kna	0.287
Kcnmb1	β1 subunit	0.127
Kcnn3	KCa2.3	0.043
Kcnt1	KCa4.1	0.019
Kcnu1	KCa5.1	0.014
Kcnn4	KCa3.1	0.008
Kcnma1	KCa1.1	0.007
Kcnmb2	β2 subunit	0.000
Kcnmb3	β3 subunit	0.000
Kcnmb4	β4 subunit	0.000
**Potassium channels, voltage-gated**		
Kcng2	Kv6.2	44.470
Kcnh2	Kv11.1	17.558
Kcnd2	Kv4.2	12.960
Kcnq1	Kv7.1	12.079
Kcnb1	Kv2.1	8.310
Kcna5	Kv1.5	4.250
Kcnv2	Kv8.2	3.502
Kcnd3	Kv4.3	3.270
Kcna7	Kv1.7	2.800
Kcne1	KCNE1	1.917
Kcnq4	Kv7.4	1.326
Kcna4	Kv1.4	0.907
Kcne4	KCNE4	0.833
Kcnf1	Kv5.1	0.000
Kcnc3	Kv3.3	0.566
Kcnc1	Kv3.1	0.237
Kcna1	Kv1.1	0.175
Kcne2	KCNE2	0.005
Kcna6	Kv1.6	0.119
Kcnab3	KCAB3	0.113
Kcna2	Kv1.2	0.105
Kcnab2	KCAB2	0.088
Kcnab1	KCAB1	0.056
Kcns1	Kv9.1	0.051
Kcnq5	Kv7.5	0.048
Kcnc4	Kv3.4	0.041
Kcnd1	Kv4.1	0.031
Kcnc2	Kv3.2	0.029
Kcnh1	Kv10.1	0.027
Kcng4	Kv6.4	0.020
Kcna3	Kv1.3	0.019
Kcns3	Kv9.3	0.012
Kcnh3	Kv12.2	0.008
Kcnh6	Kv11.2	0.007
Kcng3	Kv6.3	0.005
Kcne3	KCNE3	0.060
Kcnq3	Kv7.3	0.004
Kcnb2	Kv2.2	0.003
Kcnq2	Kv7.2	0.002
Kcnh8	Kv12.1	0.002
Kcna10	Kv1.8	0.000
Kcng1	Kv6.1	0.000
Kcnh4	Kv12.3	0.000
Kcnh5	Kv10.2	0.000
Kcnh7	Kv11.3	0.000
Kcns2	Kv9.2	0.000
Kcnv1	Kv8.1	0.000
**Potassium channels, inwardly rectifying**		
Abcc9	SUR2A,SUR2B	85.126
Kcnj11	Kir6.2	51.960
Kcnj3	Kir3.1	26.920
Kcnj5	Kir3.4	24.795
Kcnj2	Kir2.1	20.573
Kcnj12	Kir2.2	12.050
Kcnj8	Kir6.1	8.852
Abcc8	SUR1	8.225
Kcnj14	Kir2.4, Kir1.3	0.620
Kcnj4	Kir2.3	0.322
Kcnj15	Kir4.2	0.105
Kcnj9	Kir3.3	0.016
Kcnj1	Kir1.1	0.003
Kcnj10	Kir4.1, Kir1.2	0.002
Kcnj13	Kir7.1, Kir1.4	0.000
Kcnj16	Kir5.1	0.000
Kcnj6	Kir3.2	0.000
**Potassium channels, two-P**		
Kcnk3	K2P3.1, TASK-1	76.710
Kcnk6	K2P6.1, TWIK-2	0.580
Kcnk1	K2P1.1, TWIK-1	0.280
Kcnk5	K2P5.1, TASK-2	0.104
Kcnk2	K2P2.2, TREK-1	0.078
zKcnk13	K2P13.1, THIK-1	0.066
Kcnk7	K2P7.1	0.007
Kcnk10	K2P10.1, TREK-2	0.006
Kcnk12	K2P12.1, THIK-2	0.000
Kcnk15	K2P15.1, TASK-5	0.000
Kcnk16	K2P16.1, TASLK-1	0.000
Kcnk18	K2P18.1, TRESK-2	0.000
Kcnk4	K2P4.1, TRAAK	0.000
Kcnk9	K2P9.1, TASK-3	0.000
**Hydrogen voltage-gated ion channels**		
Hvcn1	Hv1	0.138
Voltage-gated ion channel genes, their respective proteins, and transcript per million (TPM) values averaged from five WT samples.		

**Table 2:** Expression of ligand-gated ion channels

**Gene**	**Protein**	**TPM**
**5-HT (serotonin) receptors, ionotropic**		
Htr3a	5-HT3A	0.023
Htr3b	5-HT3B	0.000
**Acetylcholine receptors, nicotinic**		
Chrna2	ACHA2	1.073
Chrnb1	ACHB	0.668
Chrnb2	ACHB2	0.066
Chrng	ACHG	0.028
Chrna10	ACH10	0.019
Chrna1	ACHA	0.014
Chrne	ACHE	0.007
Chrna5	ACHA5	0.003
Chrna3	ACHA3	0.000
Chrna4	ACHA4	0.000
Chrna6	ACHA6	0.000
Chrna7	ACHA7	0.000
Chrna9	ACHA9	0.000
Chrnb3	ACHB3	0.000
Chrnb4	ACHB4	0.000
Chrnd	ACHD	0.000
**GABA(A) receptors**		
Gabrr2	GBRR2	0.855
Gabra3	GBRA3	0.155
Gabre	GBRE	0.102
Gabrb3	GBRB3	0.058
Gabrq	GBRT	0.022
Gabrg3	GBRG3	0.021
Gabrb2	GBRB2	0.019
Gabra2	GBRA2	0.006
Gabrd	GBRD	0.004
Gabra5	GBRA5	0.004
Gabrg1	GBRG1	0.002
Gabra4	GBRA4	0.002
Gabra1	GBRA1	0.002
Gabra6	GBRA6	0.000
Gabrb1	GBRB1	0.000
Gabrg2	GBRB2	0.000
Gabrp	GBRP	0.000
Gabrr1	GBRR1	0.000
Gabrr3	GBRR3	0.000
**Glutamate receptors, ionotropic**		
Grik5	GRIK5	0.830
Grin2c	NMDE3	0.407
Grin3b	NMD3B	0.151
Gria3	GRIA3	0.148
Grin2d	NMDE4	0.106
Gria1	GRIA1	0.022
Grik4	GRIK4	0.018
Grik3	GRIK3	0.016
Grik2	GRIK2	0.010
Grin3a	NMD3A	0.008
Grin2a	NMDE1	0.006
Gria4	GRIA4	0.005
Grid2	GRID2	0.003
Gria2	GRIA2	0.002
Grid1	GRID1	0.001
Grik1	GRIK1	0.001
Grin1	NMDZ1	0.001
Grin2b	NMDE2	0.000
**Glycine receptors**		
Glra4	GLRA4	0.045
Glra1	GLRA1	0.000
Glra2	GLRA2	0.000
Glra3	GLRA3	0.000
**Purinergic receptors, ionotropic**		
P2rx5	P2X5	8.390
P2rx4	P2X4	2.570
P2rx6	P2X6	1.069
P2rx7	P2X7	0.349
P2rx3	P2X3	0.191
P2rx1	P2X1	0.065
P2rx2	P2X2	0.010
**Zinc-activated channels**		
*not expressed in mice*		
Ligand-gated ion channel genes, their respective proteins, and transcript per million (TPM) values averaged from five WT samples.		

**Table 3:** Expression of other ion channels

**Gene**	**Protein**	**TPM**
**Acid-sensing (proton-gated) ion channels**		
Asic3	ASIC3	0.068
Asic1	ASIC1	0.053
Asic4	ASIC4	0.009
Asic2	ASIC2	0.000
**Aquaporins**		
Aqp1	AQP1	82.978
Aqp7	AQP7	5.290
Aqp8	AQP8	4.274
Aqp4	AQP4	2.601
Aqp11	AQP11	0.154
Aqp6	AQP6	0.047
Aqp2	AQP2	0.043
Aqp5	AQP5	0.018
Aqp9	AQP9	0.007
Aqp12	AQP12	0.000
Aqp3	AQP3	0.000
Mip	MIP	0.000
**Chloride channels, voltage-sensitive**		
Clcn4	CLCN4	15.442
Clcn7	CLCN7	8.289
Clcn3	CLCN3	5.871
Clcn6	CLCN6	2.146
Clcn1	CLCN1	1.737
Clcn2	CLCN2	0.667
Clcnkb	CLCKB	0.550
Clcn5	CLCN5	0.216
Clcnka	CLCKA	0.003
**Cystic fibrosis transmembrane conductance regulators**		
Cftr	CFTR	0.006
**Calcium-activated chloride channels**		
Ano10	ANO10	12.374
Ano8	ANO8	4.659
Best3	BEST3	4.218
Ano4	ANO4	1.961
Ano1	ANO1	1.565
Ano5	ANO5	1.432
Ano6	ANO6	1.228
Ano3	ANO3	0.033
Best1	BEST1	0.016
Ano9	ANO9	0.011
Best2	BEST2	0.006
Ano2	ANO2	0.000
Ano7	ANO7	0.000
**Chloride intracellular channels**		
Clic4	CLIC4	72.409
Clic5	CLIC5	65.201
Clic1	CLIC1	10.695
Clic3	CLIC3	0.398
Clic6	CLIC6	0.039
**Gap junction proteins**		
Gja1	CXA1	82.934
Gja3	CXA3	7.013
Gjc1	CXG1	2.952
Gja4	CXA4	1.946
Gja5	CXA5	0.613
Gja6	CXA6	0.147
Gjc2	CXG2	0.095
Gjd3	CXD3	0.069
Gjb5	CXB5	0.019
Gjc3	CXG3	0.015
Gjb2	CXB2	0.005
Gja10	CXA10	0.000
Gja8	CXA8	0.000
Gjb1	CXB1	0.000
Gjb3	CXB3	0.000
Gjb4	CXB4	0.000
Gjb6	CXB6	0.000
Gjd2	CXD2	0.000
Gjd4	CXD4	0.000
Gje1	GJE1	0.000
**IP3 receptors**		
Itpr1	ITPR1	4.382
Itpr2	ITPR2	1.929
Itpr3	ITPR3	0.798
**Pannexins**		
Panx2	PANX2	0.207
Panx1	PANX1	0.137
Panx3	PANX3	0.000
**Ryanodine receptors**		
Ryr2	RYR2	225.160
Ryr3	RYR3	0.220
Ryr1	RYR1	0.047
**Sodium leak channels, non-selective**		
Nalcn	NALCN	0.049
**Sodium channels, non-voltage-gated**		
Scnn1a	SCNNA	0.077
Scnn1b	SCNNB	0.000
Scnn1g	SCNNG	0.000
Other ion channel genes, their respective proteins, and transcript per million (TPM) values averaged from five WT samples.		

**Table 4:** RNA-seq raw data and mapping metrics.

**Sample ID**	**Genotype**	**# read pairs total**	**# non-rRNA read pairs**	**% of total**	**Insert size**	**# read pairs mapping to a gene**	**% of total**	**# no-feature read pairs**	**% of total**	**# ambiguous read pairs**	**% of total**
CASK_Ct1	WT+Cre	47,543,799	47,343,548	99.58	492	34,076,980	71.67	2,721,942	5.73	8,287,647	17.43
CASK_Ct2	WT+Cre	45,437,500	45,287,356	99.67	476	33,988,592	74.8	1,440,229	3.17	7,578,641	16.68
CASK_Ct3	WT+Cre	55,117,414	54,944,721	99.69	479	40,469,641	73.42	1,790,829	3.25	11,381,005	20.65
CASK_KO1	CASK_fl+Cre	45,685,573	45,565,815	99.74	472	32,765,612	71.72	5,738,568	12.56	5,504,670	12.05
CASK_KO2	CASK_fl+Cre	55,895,769	55,607,105	99.48	511	39,344,558	70.39	2,372,238	4.24	12,476,403	22.32
CASK_KO3	CASK_fl+Cre	56,437,329	56,008,449	99.24	499	42,159,185	74.7	2,804,256	4.97	9,655,232	17.11
MDX_1	MDX	17,485,935	17,320,513	99.05	380	*(sample exluded)*					
MDX_2	MDX	39,536,744	39,037,330	98.74	447	27,475,113	69.49	3,258,092	8.24	6,543,268	16.55
MDX_3	MDX	39,626,959	39,432,254	99.51	455	27,841,146	70.26	2,169,924	5.48	7,327,584	18.49
MDX_4	MDX	42,406,246	40,919,896	96.49	488	29,805,158	70.28	2,905,415	6.85	6,497,990	15.32
MDX_5	MDX	50,934,677	47,518,076	93.29	484	34,233,480	67.21	1,864,210	3.66	10,028,518	19.69
MDX_Ct1	WT	48,311,563	46,288,106	95.81	380	32,827,800	67.95	4,353,181	9.01	7,779,264	16.1
MDX_Ct2	WT	47,283,192	46,988,962	99.38	446	32,237,279	68.18	2,304,142	4.87	10,939,883	23.14
MDX_Ct3	WT	35,275,617	34,938,284	99.04	427	24,235,276	68.7	3,631,537	10.29	4,922,208	13.95
MDX_Ct4	WT	33,977,175	32,900,815	96.83	515	25,298,933	74.46	1,713,065	5.04	4,619,558	13.6
MDX_Ct5	WT	49,379,536	45,499,492	92.14	485	32,227,570	65.27	1,210,708	2.45	10,698,976	21.67
SAP_Ct1	WT+Cre	47,930,112	47,715,719	99.55	461	34,192,649	71.34	1,965,652	4.1	9,896,590	20.65
SAP_Ct2	WT+Cre	44,934,245	44,566,395	99.18	444	30,350,071	67.54	4,879,732	10.86	7,491,483	16.67
SAP_Ct3	WT+Cre	43,586,968	43,382,766	99.53	451	29,836,839	68.45	1,332,267	3.06	8,751,881	20.08
SAP_KO1	SAP_fl+Cre	44,319,566	44,146,526	99.61	452	34,155,235	77.07	2,606,959	5.88	5,090,692	11.49
SAP_KO2	SAP_fl+Cre	41,547,517	41,397,099	99.64	469	28,697,320	69.07	3,765,768	9.06	7,431,842	17.89
SAP_KO3	SAP_fl+Cre	46,143,349	45,812,985	99.28	443	30,710,635	66.55	5,476,238	11.87	8,174,538	17.72
Total and non-ribosomal RNA read pairs, average RNA fragment size (bp), and mapping metrics, including absolute number and percentages of read pairs mapping to all annotated exons of the mouse reference genome, and no-feature and ambiguous reads, per sample. Note the low number of read pairs in MDX_1, which is therefore excluded from further analysis. CASK KO and Ctrl, SAP97 KO and Ctrl *n*s=3, MDX KO *n*=4, MDX Ctrl *n*=5.											
