# Corrigendum: Transcriptomic analyses of murine ventricular cardiomyocytes

**DOI:** 10.1038/sdata.2018.216

**Published:** 2018-10-09

**Authors:** Morgan Chevalier, Sarah H. Vermij, Kurt Wyler, Ludovic Gillet, Irene Keller, Hugues Abriel

Correction to: *Scientific Data* https://doi.org/10.1038/sdata.2018.170, published online 21 August 2018.

In the CASK and SAP97 knockout mice sub-section of the Methods in this Data Descriptor it is incorrectly stated that CASK KO mice were generated as previously published in references 9 and 20. This is incorrect. Instead, mice in which the first coding exon of the CASK gene is flanked by *loxP* sites (CASK^tm1Sud^, purchased from the Jackson Laboratory, stock #006382) were crossed with αMHC-Cre mice.

In the same section of the Data Descriptor it is incorrectly stated that in SAP97 mice the first SAP97 gene was floxed. In actuality the first three coding exons were floxed.

In Table 4 of the Data Descriptor, the genotype of samples SAP_Ct1, SAP_Ct2 and SAP_Ct3 is incorrectly listed as WT+Cre. The correct genotype is WT_fl, consistent with Figure 2 and the Mouse models sub-section of the Methods.

